# A Physiology-Guided Classification of Active-Stress and Active-Strain Approaches for Continuum-Mechanical Modeling of Skeletal Muscle Tissue

**DOI:** 10.3389/fphys.2021.685531

**Published:** 2021-08-02

**Authors:** Thomas Klotz, Christian Bleiler, Oliver Röhrle

**Affiliations:** ^1^Chair for Continuum Biomechanics and Mechanobiology, Institute for Modelling and Simulation of Biomechanical Systems, University of Stuttgart, Stuttgart, Germany; ^2^Stuttgart Center for Simulation Sciences (SC SimTech), University of Stuttgart, Stuttgart, Germany

**Keywords:** skeletal muscle, muscle modeling, continuum mechanics, active stress, active strain

## Abstract

The well-established sliding filament and cross-bridge theory explain the major biophysical mechanism responsible for a skeletal muscle's active behavior on a cellular level. However, the biomechanical function of skeletal muscles on the tissue scale, which is caused by the complex interplay of muscle fibers and extracellular connective tissue, is much less understood. Mathematical models provide one possibility to investigate physiological hypotheses. Continuum-mechanical models have hereby proven themselves to be very suitable to study the biomechanical behavior of whole muscles or entire limbs. Existing continuum-mechanical skeletal muscle models use either an active-stress or an active-strain approach to phenomenologically describe the mechanical behavior of active contractions. While any macroscopic constitutive model can be judged by it's ability to accurately replicate experimental data, the evaluation of muscle-specific material descriptions is difficult as suitable data is, unfortunately, currently not available. Thus, the discussions become more philosophical rather than following rigid methodological criteria. Within this work, we provide a extensive discussion on the underlying modeling assumptions of both the active-stress and the active-strain approach in the context of existing hypotheses of skeletal muscle physiology. We conclude that the active-stress approach resolves an idealized tissue transmitting active stresses through an independent pathway. In contrast, the active-strain approach reflects an idealized tissue employing an indirect, coupled pathway for active stress transmission. Finally the physiological hypothesis that skeletal muscles exhibit redundant pathways of intramuscular stress transmission represents the basis for considering a mixed-active-stress-active-strain constitutive framework.

## 1. Introduction

**Figure 1:**
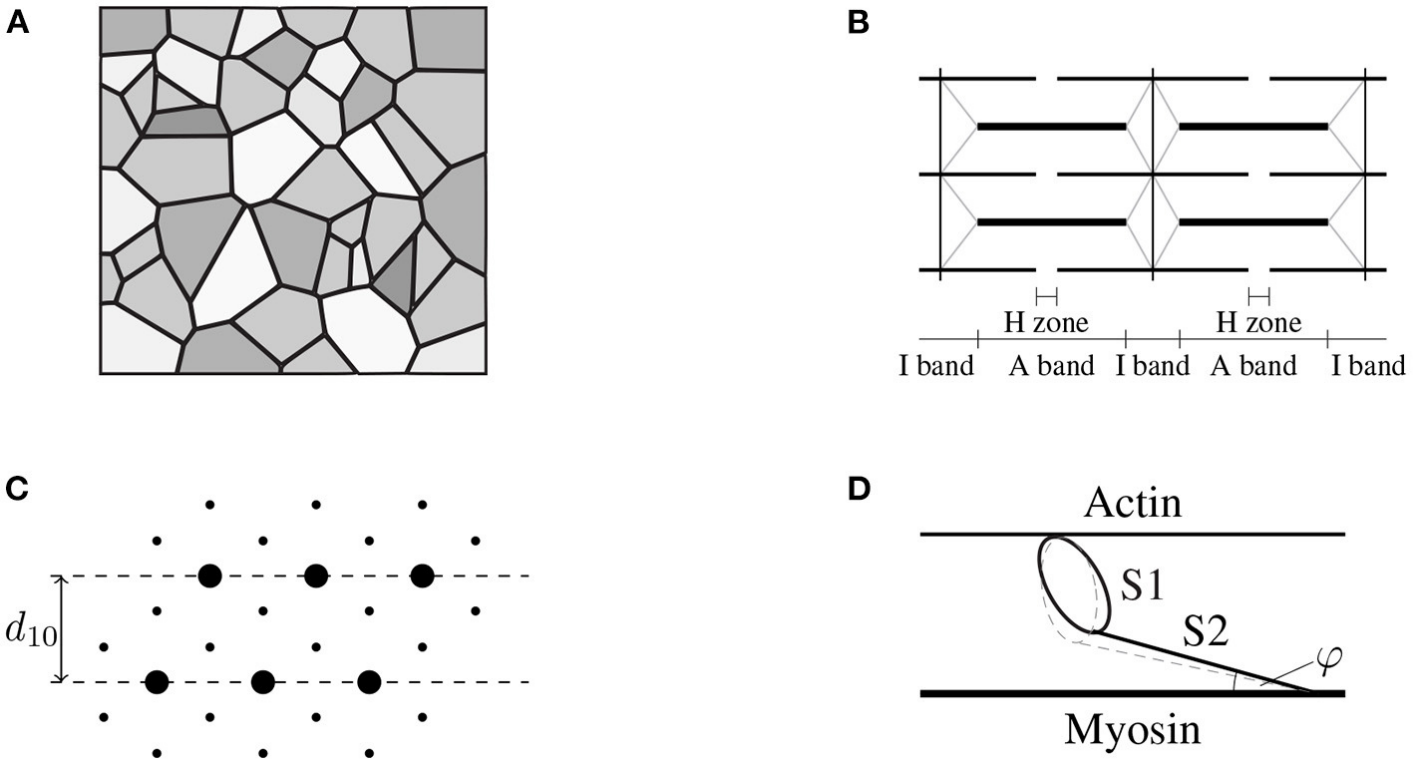
**(A)** Schematic drawing of the cross-sectional area of skeletal muscle tissue (macro-scale) consisting of muscle fibers (gray), which are aligned in parallel with the extracellular connective tissue (black). Note that the intracellular and the extracellular space are separated by the muscle fiber membrane (sarcolemma). Further note that the muscle fibers, the sarcolemma and the extracellular connective tissue are mechanically interconnected through protein complexes like dystrophin. **(B)** The muscle fibers are constructed by a periodic filament lattice (micro-scale) consisting of the thick myosin filaments (thick black lines) and the thin actin filaments (thin black lines). The individual sarcomeres are separated by the Z-disks (illustrated by the vertical black lines). Further, the Z-disks are connected to the thick myosin filaments via the protein titin (gray lines). **(C)** Illustration of a cross section of the hexagonal filament lattice (micro-scale) in the overlap region of the A-Band. The thick myosin filaments are represented by big circles, the small black dots represent the thin actin filaments. The muscle fibers are orthogonal to the page. **(D)** Schematic drawing of a single cross-bridge (nano-scale) consisting of a catalytic domain (S1-head) and the S2 segment. The S1 head can perform a conformational change (illustrated by the dashed gray line) by the cost of ATP-hydrolysis, leading either to an extension of the S1 segment, i.e., generating stress, or leading to muscle contraction.

Unlike many biological tissues, skeletal muscles have the ability to actively contract and generate mechanical stress through a complex interplay of cellular processes. Skeletal muscle tissue is a hierarchical structure mainly consisting of muscle fibers, i.e., the muscle cells, and extracellular connective tissue (cf. Figure 1). The muscle fibers itself are made up of thousands of sarcomeres (the basic contractile units), which essentially consist of a periodic lattice of thin actin and thick myosin filaments that can slide relatively to each other without changing their own length. Skeletal muscle's active behavior is closely related to its microstructure and can be explained by the sliding filament (Huxley and Hanson, 1954; Huxley and Niedergerke, 1954) and the cross-bridge (Huxley, 1957) theory. In summary, in the presence of calcium ions, which serve as a second messenger, the catalytic domain of the myosin heads (the so-called S1-segment) can bind to specialized binding sites on the thin filament. The resulting bound, which consists of a myosin head and an actin binding site, is denoted as cross-bridge. The conformational change, which myosin heads can undergo, represent the molecular motor's working stroke. Depending on the boundary conditions, the working stroke yields a relative motion between the thin and the thick filaments or an elongation of the myosin heads' molecular spring. After completing the working stroke, the cross-bridge can detach from the thin filament and returns to its initial state. The repeated process of attachment, working stroke and detachment is known as cross-bridge cycle. Thus, through cross-bridge cycling, the molecular motor converts chemically stored energy into mechanical work. While the classical two filament model captures many important features of active force generation (on the sarcomere-scale), it should be noted that further cellular proteins/structures are mechanically important for the physiological function of muscle tissue. For example, the identification of an additional myofilament named titin (Maruyama, 1976; Maruyama et al., 1977; Wang et al., 1979) within the sarcomeres of skeletal muscles refined the physiological knowledge on muscle contraction (Herzog, 2018), i.e., providing further insights how cell integrity can be ensured and enabling novel mechanistic explanations for phenomena such as force-enhancement or force depression (cf., e.g., Abbott and Aubert, 1952; Edman et al., 1982; Noble, 1992; Herzog and Leonard, 2002).

Besides principles of cellular force generation, a further important aspect of muscle physiology is how microscopically generated active stresses are balanced / transmitted through the tissue. This is important, as it is expected that intramuscular stress transmission is closely related to tissue remodeling and injury. While there is little doubt that (active) stress transmission occurs via the cytoskeleton of the muscle fibers, i.e., actin and myosin filaments, also the extracellular connective tissue plays a crucial role in efficiently transmitting locally generated stresses (Patel and Lieber, 1997; Huijing, 1999; Monti et al., 1999), for example, via activation induced along-the-fiber shear strains (Trotter et al., 1995; Purslow, 2002).

Although the physiology of skeletal muscles has been extensively studied in the past (cf. e.g., MacIntosh et al., 2006; Enoka, 2008), the current knowledge on both physiological and pathological conditions remains incomplete. Besides experimental studies, mathematical models have been established early on as valuable method to study the mechanical/physical behavior of skeletal muscles using different modeling approaches, addressing different research questions, and employing different modeling assumptions. For example, the class of microstructural motivated cross-bridge dynamics models introduced by Huxley (1957) has been used extensively to study physiological hypotheses on force production, both comprehensively and quantitatively (cf. e.g., Julian, 1969; Huxley and Simmons, 1971; Hill, 1974; Eisenberg et al., 1980; Smith, 1990; Piazzesi and Lombardi, 1995). Even before an elaborate physiological knowledge on muscle contraction had been established, Hill (1938) proposed a rheological muscle model, consisting of an elastic spring with an force generating active element in parallel. This relatively simple modeling approach can relate muscle stress, contraction velocity and energy dissipation (cf. e.g., Hill, 1938; Winters and Stark, 1987; Zajac, 1989) and thus can assist the interpretation experimental data or is frequently used to simulate motions of whole systems within multi-body modeling approaches (cf. e.g., Zajac, 1993; Anderson and Pandy, 2001; Lloyd and Besier, 2003; Delp et al., 2007). The biggest drawback of these models, however, is the fact that the model parameters cannot be attributed to specific structural elements and all spatial muscle properties are lumped.

More details on muscle function on the tissue scale can be obtained by employing three-dimensional, continuum-mechanical skeletal muscle models (based on the theory of finite elasticity), as it is possible to resolve the anatomical structure of tissues and spatial heterogeneities. Such continuum-mechanical models have, for example, been used to study intramuscular force transmission (Huijing, 1999; Yucesoy et al., 2003), the influence of the muscle fiber architecture and geometry on force output and tissue deformations (Huijing and Slawnych, 2000; Blemker and Delp, 2005; Fiorentino and Blemker, 2014; Seydewitz et al., 2019; Cankaya et al., 2021), the influence of motor unit activity and distribution (Röhrle et al., 2012; Schmid et al., 2019), or the interplay between different tissues/structures (Röhrle et al., 2017; Ramasamy et al., 2018; Pamuk et al., 2020). Further, employing the concept of classical field theories, continuum-mechanical muscle models offer great flexibility within multi-physics modeling frameworks and thus represent an important component to realize the vision of *in silico* laboratories (cf. e.g., Klotz et al., 2019; Röhrle et al., 2019; Schmid et al., 2019). While there exist numerous applications that could benefit from continuum-mechanical muscle models, the usage is still limited, for example, due to high computational cost, representation of the complex tissue geometry, mesh generation and identification of its baseline mechanical state from medical imaging data. While the latter one is of particular importance for musculoskeletal system modeling, the biggest issue for continuum-mechanical muscle modeling is the mathematical formulation of the material behavior, which is essential to obtain accurate predictions of both tissue deformations and the muscle's stress response.

To model the mechanical behavior of skeletal muscle tissues (just like for cardiac or smooth muscles) within a continuum-mechanical framework, there exists mainly two different approaches: the active-stress approach (also known as additive split) and the active-strain approach (also known as multiplicative split). While the active-stress approach derives the overall stress tensor from the linear superposition of the passive and active-stress contributions (cf. e.g., McCulloch et al., 1992; Martins et al., 1998; Nash and Hunter, 2000; Blemker et al., 2005; Röhrle et al., 2008; Heidlauf et al., 2016; Schmid et al., 2019), the active-strain approach employs a multiplicative decomposition of the deformation gradient tensor (cf. e.g., Kondaurov and Nikitin, 1987; Taber and Perucchio, 2000; Nardinocchi and Teresi, 2007; Ambrosi et al., 2011a; Ehret et al., 2011; Stålhand et al., 2011; Hernández-Gascón et al., 2013; Giantesio and Musesti, 2017; Seydewitz et al., 2019) to incorporate the muscles' active behavior. Any continuum-mechanical formulation, however, is (almost) worthless without appropriate experimental data that characterize the mechanical behavior of the material itself and which is essential for accurately calibrating a specific constitutive model. Thereby note that muscle tissue is highly diverse and suitable calibration data even for an individual muscle is just partially available—data might, for example, only consider uniaxial deformations (e.g., Zajac, 1989; Hawkins and Bey, 1994) or the passive state of the tissue (e.g., van Loocke et al., 2006; Böl et al., 2012; Takaza et al., 2013). Thus, active discussions about which approach is more suitable to incorporate active behavior seems to be more philosophical than based on rigorous validation. Rather methodological approaches are given by Ambrosi and Pezzuto (2012) and Rossi et al. (2012). They compared the additive split and the multiplicative split by focusing on mathematical aspects and concluded that the active-strain approach might be beneficial, as it is then straightforward to ensure convexity. This, in turn, guarantees the existence of a unique solution—a highly beneficial mathematical feature. Further, Giantesio et al. (2019) compared the active-stress and the active-strain approach for generic active materials and pointed out clearly that the two approaches are mechanically distinct and only coincide when rather restrictive constraints are applied. However, those studies do not provide any insights about the appropriateness of the respective model to capture the mechanical behavior of skeletal muscle tissue.

Within this work, we address this shortcoming by providing a discussion on existing constitutive modeling frameworks, i.e., the active-stress and the active-strain approach, in the context of physiological hypothesis of skeletal muscle function. To illustrate the major ideas within this work, we utilize distinct rheological models as well as specific examples for each modeling approach. We conclude that with respect to intramuscular stress transmission both the additive and the multiplicative split represent extreme scenarios. That is, while the active-stress approach follows the assumption that active and passive muscle stresses are independent and thus must be balanced within separate structural components, the active-strain approach assumes that activation yields a microscopic configuration change within the tissue and thus reflects a strong coupling of the active and the passive behavior. As current physiological hypothesis suspect a complex interplay of both pathways, it seems to be natural to consider a mixed-active-stress-active-strain formalism.

## 2. Methods

### 2.1. Continuum-Mechanical Modeling of Muscle Tissue

The fundamental governing equation of continuum mechanics can be derived from classical mechanics, where Newton's laws imply the conservation of linear momentum. In a continuum-mechanical approach, a local linear momentum balance is formulated at each material point, parametrized by a referential vector ***X*** ∈ Ω, in the continuum body Ω. It reads

(1) $$\begin{array}{l} \rho_{0}\ddot{x}\;=\text{ Div}\left(P\right)+\rho_{0}b,\text{ in }\Omega , \end{array}$$

where ρ_0_ is the mass density in the undeformed reference configuration, ***x*** is the position of a material point in the actual configuration, ***P*** is the first Piola-Kirchhoff (nominal) stress tensor, ***b*** is a vector of body forces, and Div(·) is the referential divergence operator. Moreover, we introduce the deformation gradient tensor ***F*** = Grad(***x***) to describe the motion of the continuum body from the reference to the actual configuration, whereby Grad(·) denotes the referntial gradient operator. Now, the key is to determine the stress tensor ***P*** such that it properly reflects the mechanical behavior of the respective tissue. Otherwise, the predictive power of the continuum-mechanical model is minimal.

The starting point for any constitutive model describing the overall mechanical material behavior of skeletal muscle tissue is the mathematical description of the passive material properties. Since the physiological working range of muscles includes large deformations, the framework of finite elasticity is chosen. The dominant passive material properties originate from the complex interplay between muscle fibers and the extracellular connective tissue, whereby viscous effects are small and are therefore neglected within this work. Thus, we proceed with the framework of finite hyperelasticity and outline the fundamentals in the following. Within the concept of hyperelasticity, the material behavior is fully characterized by a volume-specific strain-energy function *W*(***F***). An indispensable requirement of constitutive material modeling is the frame indifference of the strain energy, i.e.,

(2) $$\begin{array}{l} W\left(F\right)\;=\;W\left(QF\right)\;\forall Q\;\in \text{ SO}\left(3\right), \end{array}$$

whereby ***Q*** is an arbitrary orthogonal rotation matrix and SO(3) is the special orthogonal group of dimension 3. For the sake of generality, we consider arbitrary anisotropic material properties, described by the material symmetry group MG, and require

(3) $$\begin{array}{l} W\left(F\right)\;=\;W\left(FQ^{T}\right)\;\forall Q\;\in \text{ MG }\subseteq \text{O}\left(3\right), \end{array}$$

where *O*(3) is the full orthogonal group. Material symmetry is usually accounted for by enriching the argument list of *W* by an appropriate number of structural tensors ***M***_*i*_ such that *W*(***F***) = *W*(***F***, ***M***_*i*_), cf. Boehler (1977). Note that due to the distinct arrangement of muscle fibers, skeletal muscle tissue is often assumed to be transversely isotropic. For this specific case, the material symmetry group MG contains matrices associated with arbitrary rotations around the muscle fiber axis and the structural tensors have to be invariant with respect to such transformations.

The first Piola-Kirchhoff stress tensor is straightforwardly derived from the hyperelastic potential *W* by applying the Doyle-Ericksen theorem (Simo and Marsden, 1984), yielding

(4) $$\begin{array}{l} P\left(F\right)\;=\;\frac{\partial W\left(F\right)}{\partial F}. \end{array}$$

As muscle tissue is usually modeled as incompressible material, an additional constraint *J* = det(***F***) = 1 enters the stress tensor ***P*** via a Lagrange multiplier *p* (which can be interpreted as a hydrostatic pressure). Subsequently, the first Piola-Kirchhoff stress tensor for incompressible materials is given by

(5) $$\begin{array}{l} P\left(F\right)\;=\;\frac{\partial W\left(F\right)}{\partial F}-pJF^{-T}, \end{array}$$

where ***F***^−*T*^ is the transposed inverse of ***F***. As the material properties of muscle tissue can strongly vary even within the same muscle type and the same subject, the appropriate choice of strain-energy function for a specific muscle remains a big challenge. This difficulty is also reflected by the myriad of hyperelastic strain-energy functions published for soft biological tissues and muscle tissues, see e.g., Chagnon et al. (2015).

**Figure 2:**
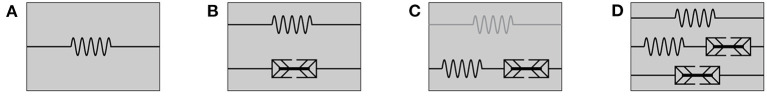
Rheological models for **(A)** passive hyperelastic behavior, **(B)** the active-stress approach, **(C)** the active-strain approach, whereby an addition of a parallel elastic element (illustrated in gray) yields a generalized active-strain approach, and **(D)** the mixed-active-stress-active-strain approach.

Furthermore, we note that the rheology of the passive material behavior presented so far can be described by an elastic spring. A schematic representation of this model is shown in Figure 2A. If, besides to the passive behavior of the muscle, the active behavior is also to be described, different methods can be used. Two existing approaches are introduced in the following, namely the active-stress approach in section 2.2 and the active-strain approach section 2.3. Further, both approaches are combined in section 2.4 to obtain a mixed-active-stress-active-strain approach. We will show that those methods correspond to distinct rheological models, in which the active behavior is described by an active element (in addition to the elastic spring for the passive behavior) and each rheological component can be associated with specific physical phenomena.

### 2.2. The Active-Stress Approach

In a continuum-mechanical framework, the most common way to model the active properties of skeletal muscle tissue is known as the active-stress approach (cf. e.g., Martins et al., 1998; Johansson et al., 2000; Blemker et al., 2005; Röhrle et al., 2008; Heidlauf et al., 2016; Schmid et al., 2019). The active-stress approach can be considered as a generalization of the pioneering muscle model of Hill (1938). Thereby, the passive material behavior is described in terms of a hyperelastic strain-energy function *W* = *W*(***F***, ***M***_*i*_). The active material behavior is modeled by adding to the passive stress that is derived from (5) a stress tensor ***P***_a_, which summarizes the microscopically generated active stresses of the muscle fibers. The overall stress tensor is then obtained as

(6) $$\begin{array}{l} P\left(F,M_{i},y\right)\;=\;P_{\text{p}}\left(F,M_{i}\right)+P_{\text{a}}\left(F,M_{i},y\right)-pJF^{-T},\\ \text{ where }P_{\text{p}}\left(F,M_{i}\right)=\frac{\partial W\left(F,M_{i}\right)}{\partial F}. \end{array}$$

Therein, the last term accounts for the incompressibility of the muscle tissue, cf. Equation (5), and a variable number of structural tensors ***M***_*i*_ accounts for general anisotropy of the passive and active parts. Further, ***y*** denotes a vector of state variables and describes the muscle's active properties. It can depend on mechanical quantities such as the macroscopic deformation ***F*** and the rate of deformation $\dot{F}$. Depending on the level of systemic detail captured by the model, the vector ***y*** can, for example, contain a set of phenomenological parameters (cf. section 2.2.2) or a set of microscopic, physically meaningful parameters that describe, e. g., the state of a population of cross-bridges (cf. section 2.2.3). Note that independent of the specific parametrization, it is assumed that the active state vector ***y*** can be externally controlled by an experimentalist and thus reflects an observable variable.

In a rheological model, the active-stress approach is described by the parallel arrangement of an elastic element and a newly introduced active element, see Figure 2B. This is necessary as the active-stress-generating property of skeletal muscles cannot be classified in terms of basic rheological elements. It should be noted that the active element, which depends on the active state vector ***y***, could have very complex constitutive properties: for example, it exhibits properties of elastic springs (e.g., reflecting short range stiffness Rack and Westbury, 1974) as well as dissipative dampers (e.g., reflecting the force-velocity relation Hill, 1938; Heidlauf et al., 2017). For a better understanding, a physiological interpretation of the active-stress approach based on microstructural considerations and some illustrative examples of specific active-stress tensors are provided in the following.

#### 2.2.1. The Active-Stress Approach in the Context of Muscle Physiology

Recalling the basic ideas of the sliding filament theory and the cross-bridge theory, the microstructural arrangement of the sarcomeres (cf. Figure 1B) allows the actin and the myosin filaments to slide in passive muscle tissue relative to each other without undergoing deformation. Assuming that both the actin and the myosin filaments are relatively stiff (note that in various microstructural models actin and myosin, are assumed to be rigid, e.g., Huxley, 1957), the overall sarcomere stress can be obtained by adding up the stress contributions of both cross-bridges and titin. Those assumptions yield an additive split of a muscle fiber stress tensor. Further, following the assumption that there is no elastic coupling between muscle fibers and the extracellular matrix, the linear superposition of the stress tensor is also justified for a tissue sample.

#### 2.2.2. Example 1: A Phenomenological Description of the Active-Stress Tensor

Assuming that the active muscle stresses only act along the fiber direction, which is indicated by a referential unit vector $a_{0}^{\text{f}}$, a corresponding active-stress tensor ***P***_a_ can be introduced as

(7) $$\begin{array}{l} P_{\text{a}}\left(F,a_{0}^{\text{f}},y\right)\;=\;P_{\text{a}}\left(y\right)F\left[a_{0}^{\text{f}}⊗a_{0}^{\text{f}}\right], \end{array}$$

where *P*_a_(***y***) reflects a scalar active-stress value. Note that the dyadic product, $a_{0}^{\text{f}}⊗a_{0}^{\text{f}}$, of the unit vector $a_{0}^{\text{f}}$ describes an appropriate structural tensor for the case of transverse isotropy and thereby replaces the general argument ***M***_*i*_. Macroscopic experiments indicate that the active muscle stress response depends on the applied stimulus, the applied stretch (Gordon et al., 1966) and the speed of the contraction (Hill, 1938). Those observations can, for example, be summarized by the following phenomenological definition of the active-stress value *P*_a_, i.e.,

(8) $$\begin{array}{l} P_{\text{a}}\left(y\right)\;=\;P_{\text{max}}f_{\text{l}}\left(\lambda_{\text{f}}\right)f_{\text{v}}\left({\dot{\lambda }}_{\text{f}}\right)\alpha \left(t\right). \end{array}$$

Therein the active state of the muscle is fully characterized by an ensemble of four scalar parameters / functions, i.e., $y={\left[P_{\text{max}},f_{\text{l}}\left(\lambda_{\text{f}}\right),f_{\text{v}}\left({\dot{\lambda }}_{\text{f}}\right),\alpha \left(t\right)\right]}^{T}$, where *P*_max_ is the maximum isometric tension and α(*t*) ∈ [0, 1] is a lumped activation parameter depending on the time *t*. The function *f*_l_(λ) denotes the force-length relation, i.e., depending on fiber stretch λ_f_, which is related to the deformation gradient tensor ***F*** and the referential fiber direction vector, $a_{0}^{\text{f}}$, by

(9) $$\begin{array}{l} \lambda_{\text{f}}^{2}\;=\;Fa_{0}^{\text{f}}\cdot Fa_{0}^{\text{f}}. \end{array}$$

Further, $f_{\text{v}}\left({\dot{\lambda }}_{\text{f}}\right)$ denotes the force-velocity relation depending on the muscle's contraction velocity $\dot{\lambda_{\text{f}}}$. It is important to note that, from a microstructural point of view, the force-length relation indicates the overlap of the actin and the myosin filaments, which is linearly proportional to the number of available cross-bridges for force generation, and therefore should not be interpreted as a hyperelastic stress-stretch relation. Thus, the active-stress tensor is only weakly coupled to the applied deformation field. Further note that a multi-axial active-stress tensor can be obtained straightforwardly by linear superposition of a set of active-stress tensors given by Equation (7), whereby one has to consider appropriate structural tensors as it is done to model, for example, the complex fiber architecture of the tongue, see, e.g., Wang et al. (2013).

#### 2.2.3. Example 2: A Microstructurally Based Description of the Active-Stress Tensor

Based on the ideas of the sliding filament theory and taking into account a skeletal muscle's microstructure, one can derive an active-stress tensor by homogenizing the microscopic cross-bridge stresses. To do so, we consider a population of *N*_xb_ cross-bridges, whereby each cross-bridge is characterized by its molecular spring's elongation *x*_*i*_. When assuming that both the actin and the myosin filament are (nearly) rigid, the overall stress tensor can be calculated, summing up the stresses of parallel arranged cross-bridges (cf. Huxley, 1957). Further one needs to take into account the periodic, microstructural geometry of skeletal muscles (cf. Figure 1). Assuming that cross-bridges behave linear elastic, the mechanical energy stored in all cross-bridges within a given reference volume $V_{\text{ref}}$ reads

(10) $$\begin{array}{l} W_{\text{active}}^{\text{xb}}\;=\;\overset{N_{\text{xb}}}{\underset{i=1}{\sum }}\frac{1}{2}k_{\text{xb}}x_{i}^{2}, \end{array}$$

where *k*_xb_ is the cross-bridge stiffness. The (scalar-valued) force produced by a single cross-bridge $F_{i}^{\text{xb}}$ can now be derived from a partial derivative of the cross-bridge energy with respect to the cross-bridge elongation *x*_*i*_, i.e.,

(11) $$\begin{array}{l} F_{i}^{\text{xb}}=k_{\text{xb}}x_{i}. \end{array}$$

Note that the cross-bridge elongations are microscopic quantities and are not directly related to the macroscopic strains. To obtain a continuum description of the active-stress tensor, the discrete cross-bridge forces need to be homogenized. When assuming that cross-bridges do not transmit bending moments or shear forces, the mechanical problem is purely geometric (Schoenberg, 1980a) and thus only the three-dimensional structure of the sarcomere needs be taken into account. Due to the periodic structure of the filament lattice (cf. Figure 1), a single half-sarcomere can be considered as representative unit cell. Using the assumption that for the considered population all cross-bridges act in parallel, the active stress in muscle fiber direction can be calculated by Schoenberg (1980b).

(12) $$\begin{array}{l} P_{\text{a}}^{\text{f}}\left(F,a_{0},y\right)\;=\;\frac{\cos\left(\varphi \right)}{A_{\text{f}}^{\text{ref}}}\overset{N_{\text{xb}}}{\underset{i=1}{\sum }}k_{\text{xb}}x_{i}F\left[a_{0}^{\text{f}}⊗a_{0}^{\text{f}}\right]. \end{array}$$

Therein $A_{\text{f}}^{\text{ref}}$ is the cross-sectional area of the reference volume and φ is the angle of force transmission (cf. Figure 1D). Due to the filament arrangement (cf. Figure 1C), the cross-fiber stresses act in hexagonal plane, i.e., which describes the filament lattice unit cell consisting of six actin filaments with one myosin filament in the center, normal to the muscle fiber direction. Thereby it is assumed that all cross-bridges are equally distributed between the thin filaments, i.e., $N_{\text{actin}}^{n}/N_{\text{xb}}^{\text{max}}=1/6$ (*n* = 1, 2, ..., 6). Hence, for each subset of cross-bridges pointing toward the same actin filament an individual stress tensor can be derived (cf. Heidlauf et al., 2016), i.e.,

(13) $$\begin{array}{l} P_{\text{a}}^{\text{xf},n}\left(F,a_{0}^{\text{f}},y\right)\;=\;\frac{1}{6}\frac{\sin\left(\varphi \right)}{A_{\text{xf}}^{\text{ref}}}\overset{N_{\text{xb}}}{\underset{i=1}{\sum }}k_{\text{xb}}x_{i}F\left[t_{n}⊗t_{n}\right],\\ n=1,2,...,6, \end{array}$$

where $A_{\text{xf}}^{\text{ref}}$ is one sixth of the mantle surface of the reference volume and the unit vectors ***t***_*n*_ are defined by

(14) $$\begin{array}{l} t_{n}\;=\;\cos\left(\phi +\left[n-1\right]\frac{\pi }{3}\right)e_{2}\\ \;+\sin\left(\phi +\left[n-1\right]\frac{\pi }{3}\right)e_{3},n=1,2,...,6\;. \end{array}$$

Therein, the orthonormal basis vectors ***e***_*i*_ (*i* = 1, 2, 3) denote a laboratory frame of reference that is–without loss of generality–defined such that $e_{1}=a_{0}^{\text{f}}$. Further, the angle ϕ ∈ [0, 2π) is introduced to account for the non-uniqueness of the basis vectors ***e***_2_ and ***e***_3_. The overall cross-fiber stress tensor, $P_{\text{a}}^{\text{xf}}$, is derived by adding up all the individual stresses, $P_{\text{a}}^{\text{xf,n}}$, and utilizing the fact that $\sum_{n}t_{n}⊗t_{n}\;=3\left(I-\left(a_{0}^{\text{f}}⊗a_{0}^{\text{f}}\right)\right)$, (cf. Usyk et al., 2000; Heidlauf et al., 2016), yielding

(15) $$\begin{array}{r} P_{\text{a}}^{\text{xf}}\left(F,a_{0}^{\text{f}},y\right)\;=\;\overset{6}{\underset{n=1}{\sum }}P_{\text{a}}^{\text{xf},n}\;=\\ \frac{1}{2}\frac{\sin\left(\varphi \right)}{A_{\text{xf}}^{\text{ref}}}\overset{N_{\text{xb}}}{\underset{i=1}{\sum }}k_{\text{xb}}x_{i}F\left[I-\left[a_{0}^{\text{f}}⊗a_{0}^{\text{f}}\right]\right]. \end{array}$$

Finally, the overall active-stress tensor ***P***_a_ is given by

(16) $$\begin{array}{l} P_{\text{a}}\left(F,a_{0}^{\text{f}},y\right)\;=\;P_{\text{a}}^{\text{f}}\left(F,a_{0}^{\text{f}},y\right)+P_{\text{a}}^{\text{xf}}\left(F,a_{0}^{\text{f}},y\right), \end{array}$$

where the active state vector is summarized by $y={\left[k_{\text{xb}},A_{\text{f}}^{\text{ref}},A_{\text{xf}}^{\text{ref}},x_{1},...,x_{N_{\text{xb}}}\right]}^{T}$. Employing a set of experimentally derived parameters (cf. Appendix), yields at optimal muscle length and full activation a nominal active stress in fiber direction of approximately 25 N cm-2. This coincides well with experimental findings for skeletal muscle tissue (Bodine et al., 1987). Further, the model predicts active stresses in the cross-fiber direction of approximately 5–10% of the active stress in fiber direction.

### 2.3. The Active-Strain Approach

Instead of employing the active-stress approach for modeling a skeletal muscle's active behavior, an active-strain approach has been used (e.g., Ehret et al., 2011; Hernández-Gascón et al., 2013; Giantesio and Musesti, 2017; Seydewitz et al., 2019). For the active-strain approach, one assumes that the deformation gradient tensor ***F*** can be multiplicatively split into an active part ***F***_a_ and an elastic contribution ***F***_e_, i.e.,

(17) $$\begin{array}{l} F\;=\;F_{\text{e}}\;F_{\text{a}}. \end{array}$$

Although this multiplicative decomposition was originally proposed for elastoplastic materials (Lee, 1969), it has also been applied to model a variety of other internal (inelastic) processes. In the field of biomechanics, for example, the concept was also employed to model tissue growth (Rodriguez et al., 1994; Ambrosi et al., 2011b). The basic idea of the multiplicative split is that the active deformation gradient tensor ***F***_a_ depicts a mapping from the reference configuration to an incompatible, stress-free intermediate configuration. In turn, the elastic deformation gradient tensor ***F***_e_ reflects a mapping from the intermediate configuration to the actual configuration. This means that the elastic deformation is given by $F_{\text{e}}=F{F_{\text{a}}}^{-1}$ and depends on the “visible” deformation and the active contribution. Subsequently, the mechanical behavior of the tissue is then described by a potential *W* = *W*(***F***_e_, ***M***_*i*_) as a function of the elastic part of the deformation. The resulting first Piola-Kirchhoff stress tensor is given by

(18) $$\begin{array}{r} P\left(F,M_{i},y\right)\;=\;P_{\text{e}}\left(F,M_{i},y\right)-pJF^{-T},\\ \text{where }P_{\text{e}}=\frac{\partial W}{\partial F_{\text{e}}}{F_{\text{a}}}^{-T}\;. \end{array}$$

Therein, $\left(\cdot \right){F_{\text{a}}}^{-T}$ denotes a pull-back transformation that is required since the derivative ∂*W*/∂***F***_e_ yields a two-point tensor relating forces in the actual configuration and area elements in the intermediate configuration, whereas the nominal stress ***P*** is a two-point tensor between actual and referential coordinates. Within a rheological setup, the active-strain approach corresponds to a serial arrangement of an elastic spring and an active element, see Figure 2C. Thereby, the deformation tensor ***F***_e_ corresponds to the deformation of the spring and the tensor ***F***_a_ describes the deformation of the active element. This serial characteristic is clearly different if compared to the setup of the active-stress approach in section 2.2. Note, in the active-strain approach one exploits the fact that the mechanical state of the material is fully characterized by the potential energy of the serial elastic element. Further, for skeletal muscles, it is common to additively split up the energy function and then apply the multiplicative split only to parts of the energy (Ehret et al., 2011; Hernández-Gascón et al., 2013). Such an approach can be considered as generalized active-strain approach (reminiscent to the generalized Maxwell model in viscoelasticity) and is illustrated in Figure 2C by the gray elastic spring. The key challenge remains the constitutive definition of an appropriate active deformation gradient tensor ***F***_a_, which is generically introduced to be parameterized by the potential *W*, the active state vector ***y*** and a sufficient number of structural tensors ***M***_*i*_ that describe the material symmetry of the internal active contraction, i.e., ***F***_a_(*W*, ***M***_*i*_, ***y***). It should be noted that while it is possible for the active-stress approach to derive the active-stress tensor directly from macroscopic observations (cf. section 2.2.2), i.e., a set of physical observable variables (cf. section 2.2.3), this is no longer true for the active-strain approach, as the active deformation gradient tensor ***F***_a_ represents an internal and thus non-observable variable. Further, due to the strong coupling between the elastic spring and the active element the chosen constitutive relation requires consideration of the assumed elastic potential. Finally note that in the case of zero activation, i.e., ***F***_a_ = ***I*** (whereby ***I*** is the second-order identity tensor), one obtains the previously defined purely passive muscle stresses (cf. Equation 5). In analogy to the investigations of the active-stress approach, the active-strain approach is discussed with respect to muscle physiology in section 2.3.1 and specific constitutive equations are exemplary provided in sections 2.3.2, 2.3.3.

#### 2.3.1. The Active-Strain Approach in the Context of Muscle Physiology

Given the serial arrangement of an elastic spring and an active element as shown in Figure 2C, the active-strain approach implies that there exists a strong coupling between elastic element and the active element. Muscle physiology teaches that muscle fibers not only generate active stress, but also actively change their shape. Further, fiber motion is constrained by the interaction with the surrounding extracellular connective tissue. Accordingly, any kinematic rearrangement in the microstructure (i.e., the muscle fibers) will yield a change in the elastic potential of a tissue sample. Thus, while the active-stress approach implied that active stresses are transmitted through the actin-myosin skeleton of the muscle fibers (cf. section 2.3.1), the active-strain approach describes a fundamentally different, indirect mechanism of active stress transmission. Considering current physiological hypotheses, the most likely candidate to indirectly transmit the active stresses is the extracellular connective tissue (e.g., Trotter et al., 1995; Patel and Lieber, 1997; Huijing, 1999; Monti et al., 1999; Purslow, 2002). Note that from a purely macroscopic perspective, it is impossible to observe such microscopic rearrangements [they will strongly depend on the microscopic architecture and boundary conditions, cf., e.g., Sharafi and Blemker (2011), and, thus, are barely possible to predict a priori]. Therefore, macroscopic modeling frameworks require a non-observble internal variable to incoporate this behavior. In this sense, it is important to stress that the active deformation gradient tensor has no unique physiological meaning. However, as illustrated by the two examples below, strongly simplified virtual tissue arrangements help to illustrate the general concept of the multiplicative decomposition of the deformation gradient tensor.

#### 2.3.2. Example 3: A Volume Preserving Active Contraction Along the Fiber Direction

**Figure 3:**
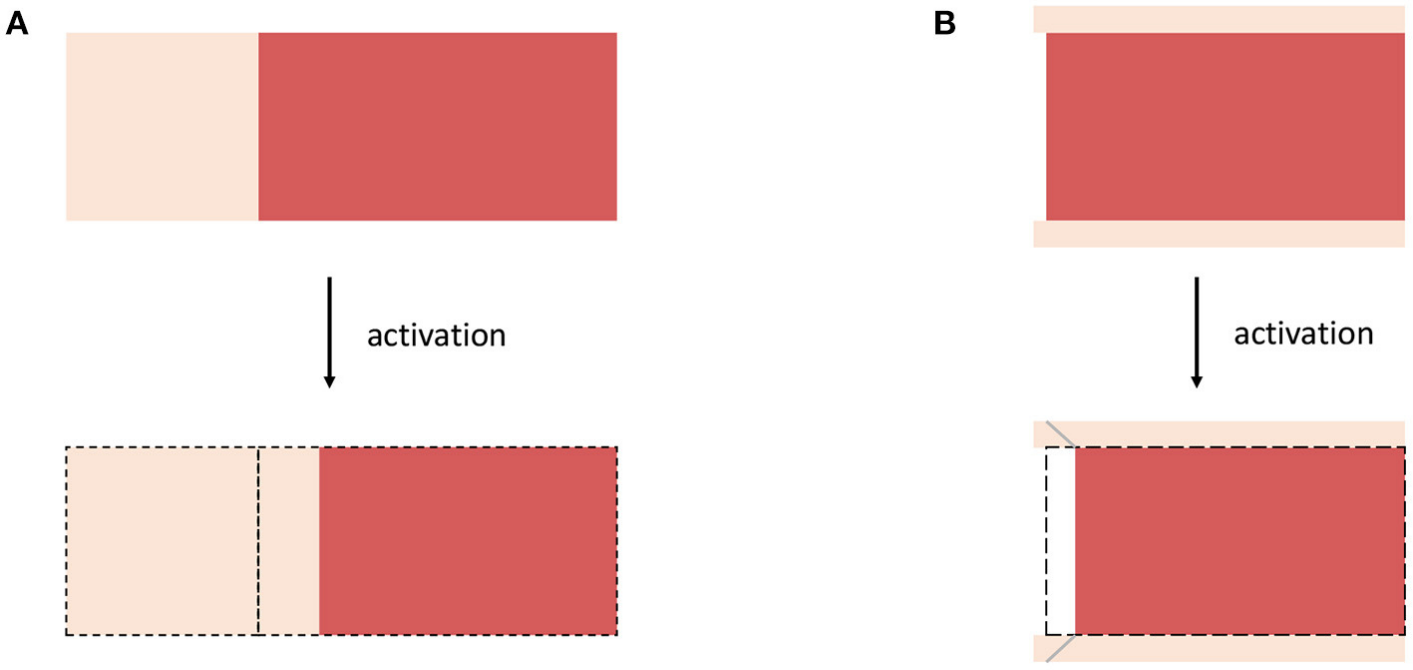
Schematic drawings for **(A)** Example 3, considering a muscle fiber (red) which is serially arranged with extracellular connective tissue (bright color) and **(B)** Example 4 considering a non-spanning muscle fiber (red) embedded in (thin) sheets of extracellular connective tissue (bright color). For both examples, the structural rearrangement caused by the activation of the muscle fibers are illustrated.

As a first example, we assume a virtual tissue sample consisting of a muscle fiber that is (serially) arrange with elastic extracellular connective tissue (cf. Figure 3A). This is similar to the experiment of Shaw et al. (2013), where a cardiac myocite is embedded within an elastic gel. Therefore, it is assumed that in response to activation the muscle fiber contracts along the fiber direction $a_{0}^{\text{f}}$ while preserving its volume, i.e., requiring det(***F***_a_) = 1. This is reflected by the active element in Figure 2C. In order to ensure compatibility with the actual configuration the serially arranged connective tissue, i.e., corresponding to the serial spring in Figure 2C, is stretched. This stretch is described by the elastic deformation gradient tensor ***F***_e_. A corresponding active deformation gradient tensor can be formulated to read

(19) $$\begin{array}{l} F_{\text{a}}\left(W,a_{0}^{\text{f}},y\right)\;=\;\lambda_{\text{a}}\left[a_{0}^{\text{f}}⊗a_{0}^{\text{f}}\right]+\frac{1}{\sqrt{\lambda_{\text{a}}}}\left[I-\left[a_{0}^{\text{f}}⊗a_{0}^{\text{f}}\right]\right]\;. \end{array}$$

Therein λ_a_ can be interpreted as a measure for the internal active stretch and reflects the internal motion/contraction of the muscle fiber. It requires an additional constitutive relation, i.e., λ_a_ = λ_a_(*W*, ***y***). Note, the active stretch λ_a_ also depends on the elastic tissue properties and therefore has to contain the potential functional *W* as argument.

#### 2.3.3. Example 4: Activation Induced Shear Strains

In this example, we assume a microstructural configuration consisting of a non-spanning muscle fiber embedded in extracellular connective tissue, which is much thinner then the diameter of the muscle fibers (cf. Figure 3B). The activation of the muscle fiber will force the fiber to contract and thus yields a relative motion between the muscle fiber and the connective tissue (note that a similar behavior is expected if active stresses are heterogeneously distributed along the length of muscle fibers). The microscopic change of the muscle fibers configurations will cause shear deformations along the fiber axis within the extracellular connective tissue. Thus, a corresponding active deformation gradient tensor can be defined to read

(20) $$\begin{array}{l} F_{\text{a}}\left(W,a_{0}^{\text{f}},y\right)\;=\;I+\gamma_{\text{a}}\left[a_{0}^{\text{f}}⊗e_{i}\right]\;. \end{array}$$

Therein, γ_a_ = γ_a_(*W*, ***y***) is the internal active shear along the fiber axis and ***e***_*i*_ denotes an arbitrary unit vector which is orthogonal with respect to the fiber direction. Note that while the scenario described in Example 3 (i.e., section 2.3.2) requires large internal motions of the muscle fibers to yield a significant stress response, for the scenario described within this section rather small changes of the muscle fiber configurations are sufficient to yield considerable change of the elastic deformation gradient tensor. This is due to the difference in the tissue components thickness, i.e., the muscle fibers are much thicker than the sheets of extracellular connective tissue separating them. Consequently, small (internal) rearrangements of the microstructure are sufficient to induce large shear deformations within the extracellular connective tissue and thus represent a very efficient pathway for active-stress transmission (cf. Trotter et al., 1995; Purslow, 2002; Sharafi and Blemker, 2011).

### 2.4. A Mixed-Active-Strain-Active-Stress Constitutive Modeling Approach

Considering stress transmission on the tissue scale, the previous sections conclude that both the active-stress and the active-strain approach physiologically represent extreme scenarios: the active-stress approach assumes a decoupled pathway for active-stress transmission (cf. section 2.2.1), while the active-strain approach is based on the assumption that the active stresses are transmitted by the coupling with an elastic matrix material (cf. section 2.3.1). Thus, for a tissue employing redundant pathways of active-stress transmission, as it is expected for skeletal muscle tissue, it seems to be natural to formulate a mixed-active-stress-active-strain constitutive modeling framework. A corresponding rheological model is illustrated in Figure 2D. Based on the constitutive equations introduced in the previous sections, cf. Equations (6), (18), we define the first Piola-Kirchhoff stress tensor as

(21) $$\begin{array}{l} P\left(F,M_{i},y\right)\;=\;\frac{\partial W_{1}\left(F,M_{i}\right)}{\partial F}+\frac{\partial W_{2}\left(F,M_{i},y\right)}{\partial F_{\text{e}}}{F_{\text{a}}}^{-T}\\ \;+P_{\text{a}}\left(F,M_{i},y\right)-pJF^{-T}. \end{array}$$

Therein, the energy *W* is additively split into contributions that depend on the overall deformation ***F*** and the partial deformation ***F***_e_. In this sense, the energy contribution that depends on ***F*** describes the parallel spring in Figure 2D, whereas the part that depends on ***F***_e_ desribes the spring that is arranged in series to the active elements (in the middle). Note that all stress components require constitutive equations, whereby one can employ the same concepts as presented in sections 2.1–2.3. Within the following section we will discuss the presented macroscopic constitutive modeling frameworks in the context of physiological hypotheses of skeletal muscle function (i.e., experimental observations) and macroscopic data from material testing, and discuss practical considerations for formulating specific constitutive models.

## 3. Results and Discussion

### 3.1. Active Constitutive Modeling Frameworks and Muscle Physiology

Considering the anatomical structure of skeletal muscles, there is little doubt that the active stresses induced by cross-bridge strains on the nano-scale can be transmitted efficiently through the muscle fiber's filament skeleton. Within a continuum-mechanical framework this aspect of active muscle behavior can be adequately modeled by introducing an active-stress tensor (cf. section 2.2). Experimental evidence for such a behavior has been derived indirectly from the observation that the maximum isometric tension and the fraction of activated muscle fibers are linearly proportional (Powell et al., 1984).

However, the existence of non-spanning muscle fibers (Huber, 1916; Lindhard, 1931; Loeb et al., 1987; Ounjian et al., 1991), i.e., muscle fibers that are not attached to a tendon on both sides and thus end in the middle of a muscle belly, clearly indicates that alternative pathways for active-stress transmission on the tissue scale must exist. Early evidence that active-stress transmission can occur via the extracellular connective tissue was provided by Ramsey and Street (1940) and Street and Ramsey (1965) showing that a preparation of an isolated muscle fiber surrounded by extracellular connective tissue is still capable of producing nearly its maximum isometric force after the filament skeleton was removed from one half of the sample. Although these experiments do not cover physiological conditions, the current view is that muscles employ redundant pathways for stress transmission (Street, 1983; Patel and Lieber, 1997; Huijing, 1999; Monti et al., 1999). Beside the obvious involvement of the muscle fiber's filament skeleton, particularly activation induced shear deformations of the extracellular connective tissue are believed to contribute to active-stress transmission (Trotter et al., 1995; Purslow, 2002). The efficiency of active-stress transmission via shear strains was emphasized by Purslow (2002) and Sharafi and Blemker (2011), pointing out that due to the small thickness of the extracellular connective tissue, modest (relative) fiber motions are sufficient to induce large shear strains; fiber motions, however, are not so uncommon. Even during isometric contractions, local sarcomere length changes, i.e., on the micro-scale, have been observed within isolated muscle fibers (Julian and Morgan, 1979) and whole muscles (Moo and Herzog, 2018). These observations on active muscle behavior can be phenomenologically captured within a macroscopic, continuum-mechanical framework by employing an active-strain approach with an internal variable (cf. section 2.3).

In conclusion, while both the active-stress and the active-strain approach seem to cover a single very specific aspect of muscle's active behavior, a more complete reflection of muscle physiology can be expected when appealing to a mixed active-stress-active-strain approach. In this connection, it can be seen that both the active-stress and the active-strain approach represent special cases of the mixed active-stress-active-strain approach, with opposing weightings for the active stress transmitted through the corresponding rheological elements.

### 3.2. Macroscopic Data and Limitations

All three approaches require a choice for specific constitutive equations. Although phenomenological constitutive equations are, unlike the governing equations of the overall system, i.e., modeling approaches, designed with the underlying material behavior and physiology in mind, they remain mathematical constructs. In this regard, constitutive parameters can not necessarily be associated with physical properties. The values of a constitutive law's “material parameters” are consequences of fitting the mathematical construct to experimentally observed data from macroscopic material experiments, i.e., from experiments that aim to relate stresses, deformations and activation. Depending on the choice of the objective function of the optimization procedure and the quality and richness of the experimental data, multiple mathematical constructs, e.g. number and exponential-, fractional-, polynomial-, or logarithmic-like terms, lead to very similar results and only a “goodness of fit” parameter, i.e., choosing an adequate norm, provides at the end a measure that favors one or the other constitutive law, cf. Schmid et al. (2007) for myocardial parameter estimation. This also applies in this setting, i.e., it is theoretically possible to replicate excellent experimental data with any of the considered constitutive frameworks; this is particularly true as the active element can capture various different mechanical properties and thus offers great flexibility. Moreover, the choice of norm to compare the different constitutive results with each other might influence the results in such a way that it is cumbersome to carry out fitting procedures for the three individual cases and compare them. This is particular true if one discusses the underlying modeling frameworks rather than the form, i.e., the individual terms, of a particular constitutive law. As the additional benefit of employing material fitting optimization procedures is marginal, we omitted within this work any material fitting procedures, and entirely focus on the advantages and disadvantages of the individual modeling approaches—in particular with respect to the underlying physiology.

Finally it must be noted that it is impossible to reconstruct the internal behavior of the tissue from purely macroscopic data. Thus, choosing the ratio between the active-stress and the active-strain contribution remains a modeler's assumption. Recalling the physiological basis of the active-stress and active-strain contributions, however, might allow an educated guess. More sophisticated choices might be possible by augmenting experimental data with data originating from multi-scale simulations, for example, by combining microstructural imaging and micro-mechanical homogenization models (e.g., Sharafi and Blemker, 2011; Bleiler et al., 2019, 2021). That is, micro-mechanical models can be used to predict microstructural tissue rearrangements and which are represented by the active deformation gradient tensor ***F***_a_ in a macroscopic modeling framework.

### 3.3. Conclusion

We want to conclude this discussion on continuum-mechanical constitutive frameworks to model muscle's active behavior by a set of practical considerations to formulate specific constitutive equations:

- For any classical continuum-mechanical boundary value problem that is relating external forces and internal deformation fields, the choice of a specific constitutive framework is rather a philosophical decision. The only limiting factor for the predictive power of an *in silico* model is the goodness of the employed macroscopic calibration data, which always can be replicated by any of the constitutive frameworks. Note, while it has been shown that the active-strain and the active-stress approach can differ for conditions that where not included in the calibration data (Giantesio et al., 2019), assessing the goodness of a predictions for such conditions is beyond the scope of any phenomenological model.
- The choice of a weighting between active-stress and active-strain components is of particular importance when employing a continuum model to investigate tissue remodeling or injuries. This is emphasized by the observation that both phenomena are determined by elastic stresses (Lieber and Friden, 1993). Thereby, one should keep in mind that various neuromuscular diseases potentially affect the elastic coupling between muscle fibers and the extracellular connective tissue.

## Data Availability Statement

The original contributions presented in the study are included in the article/supplementary material, further inquiries can be directed to the corresponding author/s.

## Author Contributions

TK and OR contributed to the conceptualization of the study. TK and CB derived the presented constitutive equations. Further, all authors contributed to the analysis and the discussion of the results as well as the writing of the manuscript.

## Conflict of Interest

The authors declare that the research was conducted in the absence of any commercial or financial relationships that could be construed as a potential conflict of interest.

## Publisher's Note

All claims expressed in this article are solely those of the authors and do not necessarily represent those of their affiliated organizations, or those of the publisher, the editors and the reviewers. Any product that may be evaluated in this article, or claim that may be made by its manufacturer, is not guaranteed or endorsed by the publisher.

## Appendix – Example 2: A Microstructurally Based Description of the Active-Stress Tensor

Predicting the active-stress tensor given in Equation (16) based on a set of physical meaningful variables, requires identification of additional model parameters related to the material behavior and the geometry of the microstructure. Therefore, a hexagonal lattice structure, i.e., where a thick (myosin) filament is surrounded by six thin (actin) filaments, is assumed (Millman, 1998). For skeletal muscle tissue the lattice spacing parameter *d*_10_ (cf. Figure 1C) is, depending on the different muscle types and species, between 35 and 45 nm (cf. Millman, 1998). Assuming *d*_10_= 36 nm and neglecting the diameter of the filaments results in a distance of *r*_am_= 24 nm between a thick and a thin filament. Further, we assume a half-sarcomere length of $l_{\text{hs}}^{\text{ref}}=$ 1 μm in the reference configuration. From the given geometrical parameters, the volume of a half-sarcomere can be calculated by

(S1) $$\begin{array}{l} V_{\text{sarco}}^{\text{ref}}=A_{\text{f}}^{\text{ref}}\cdot l_{hs}^{\text{ref}}=\frac{3}{2}\sqrt{3}\cdot r_{\text{am}}^{\text{2}}\cdot l_{\text{hs}}^{\text{ref}}=1.4965\times 10^{6}{\text{nm}}^{3}. \end{array}$$

Further, assuming that the length of the myosin filament in a half-sarcomere measures around 850 nm and exhibits a distance of about 43 nm between two myosin heads (cf. Daniel et al., 1998), then there are ~20 myosin-head complexes along one thick filament, pointing toward one thin filament. Note that one myosin-head complex consist of two head units competing for the same actin binding site. Such effects are however beyond the scope of this work and therefore neglected. Recalling the hexagonal structure, there are 6 such lines and thus the maximum number of available cross-bridges per half-sarcomere can be calculated by $N_{\text{max}}^{\text{xb}}=$ 20 · 6 = 120. During an isometric contraction and full activation ~20 to 40% of the cross-bridges are in a force producing state (Gordon et al., 2000; Brunello et al., 2014). For an exemplary calculation, we assume that for a tetanic contraction 30% of the cross-bridges are in a force producing state. Further, an average isometric power-stroke elongation of *x*_iso_= 6 nm (cf. Barclay, 1998; Brunello et al., 2014) is assumed. As far as the stiffness of the actin-myosin cross-bridges is concerned, there exists some discussion on its value. Depending on different experimental methods and theoretical assumptions, reported stiffness values range from 0.5 to 3.5 pN nm^−1^ (Barclay, 1998; Mansson et al., 2015). For the exemplary calculation presented in Section 2.2.3, we choose the stiffness of the actin-myosin cross-bridges as *k*_xb_= 2.0 pN nm^−1^. Finally, assuming that the angle of force transmission can be approximated by the length of the S2 segment *l*_S2_= 40 nm (MacIntosh, 2003) and the distance between actin filament and the myosin filament, *r*_am_, the angle of force transmission is φ ≈ 0.17π.
